# Supplementary material for: The interactome of intact mitochondria by cross-linking mass spectrometry provides evidence for coexisting respiratory supercomplexes
Source: Mol Cell Proteomics. 2017 Dec 8;17(2):216–32. doi: 10.1074/mcp.RA117.000470 (PMC5795388; doi:10.1074/mcp.RA117.000470)
Supplement: Supplemental Data [file supp_RA117.000470_133922_0_supp_23978_fzffwf.zip › spectra_annotation/mito_DR_spectra_annotation/422-1-3-1-4-1.pdf]

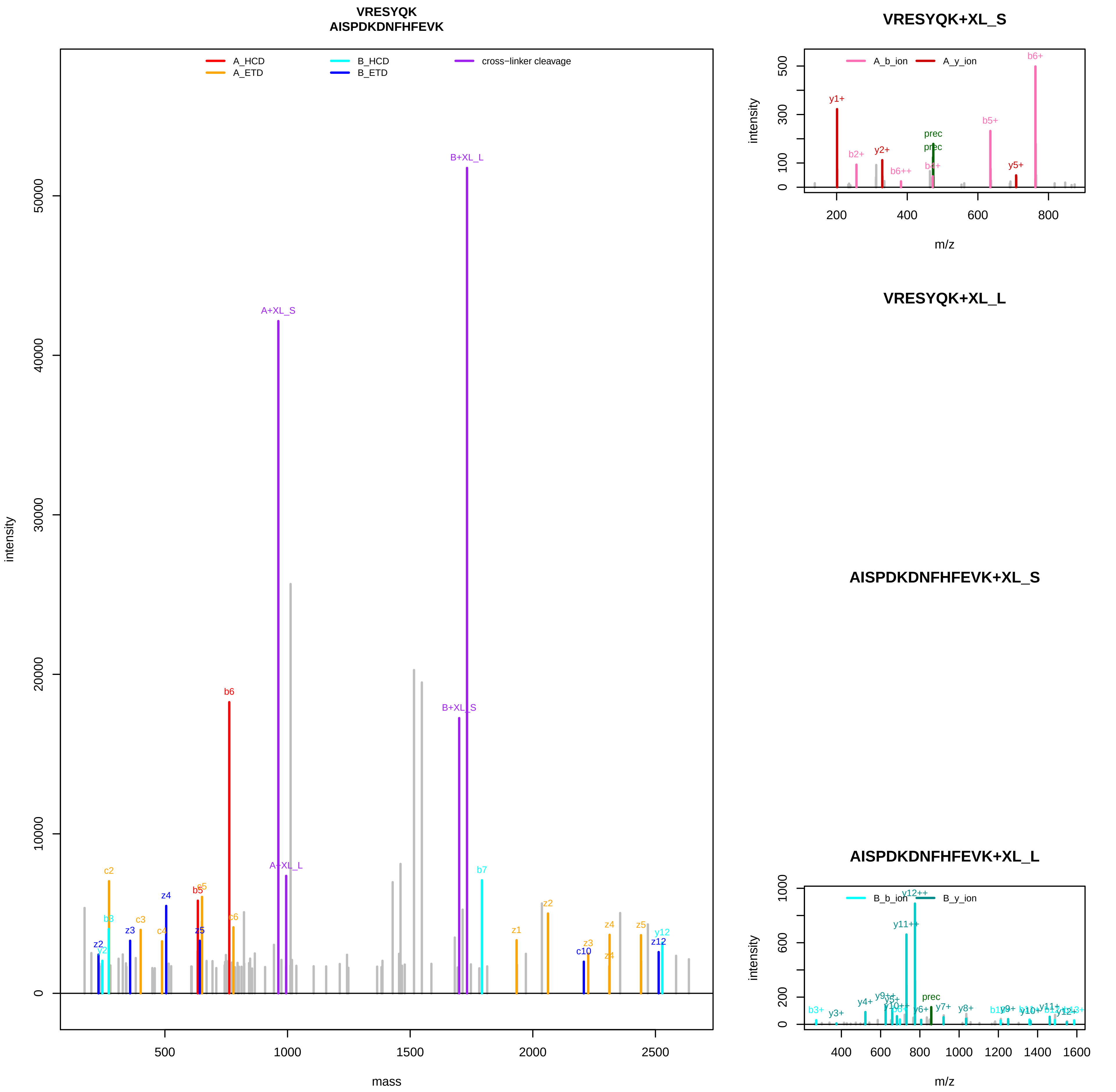
VRESYQK
AISPDKDNFHFEVK
A_HCD
A_ETD
B_HCD
B_ETD
cross−linker cleavage
B+XL_L
A+XL_S
B+XL_S
A+XL_L
b6
b5
c5
z5
c2
b3
c3
c4
z4
z3
z2
y2
c6
b7
z1
z2
c10
z3
z4
z5
y12
z12
intensity
mass
VRESYQK+XL_S
A_b_ion
A_y_ion
y1+
b2+
y2+
b6++
prec
b4+
b5+
y5+
b6+
m/z
VRESYQK+XL_L
AISPDKDNFHFEVK+XL_S
AISPDKDNFHFEVK+XL_L
B_b_ion
B_y_ion
y12++
y11++
prec
b3+
y3+
y4+
y9++
y5+
y10++
y6+
y7+
y8+
y9+
y10+
y11+
y12+
